# Supplementary material for: Vireo: Bayesian demultiplexing of pooled single-cell RNA-seq data without genotype reference
Source: Genome Biol. 2019 Dec 13;20:273. doi: 10.1186/s13059-019-1865-2 (PMC6909514; doi:10.1186/s13059-019-1865-2)
Supplement: Supplementary file 2 — Additional file 2 Review history. [file 13059_2019_1865_MOESM2_ESM.docx]

Review History

**First round of review**

**Reviewer 1**

**Comments to author:**

Vireo aims to demultiplex pooled single-cell RNA-seq experiments by cell genotyping based on scRNA-seq reads which means that reference genotypes are not required for the input samples as long as sufficient sequencing depth is achieved to allow an order of 100 common genetic variants to be typed. The Bayesian model and inference scheme are sound and the authors tackle the problem in exactly the same way I would have - I can have no complaints. The authors provide numerical experiments to demonstrate the efficacy of the method. However, these simulations and analyses only serve to provide examples of usage. The generative model used in Vireo is a reasonable model of the physical reality and I did not find the results surprising. Methodologically the paper is of publication quality.

The main uncertainty in my assessment lies in the fact that the utility of Vireo is tied substantially to the pooled experimental design and its limitations. The authors end stating that "we recommend pooling 6 to 10 samples per experiment. For extremely large pools with more than twelve samples, genotype data is still required to enable accurate de-multiplexing." It is unclear to me therefore how widely Vireo would be of interest as a result.

**Reviewer 2**

**Comments to author:**

Multiple groups have developed different tools to address this necessity, generating barcoding-based methods, for example cell-hashing and MULTI-seq, and genotype-based method represented by Demuxlet. In this manuscript, the authors propose to further develop the genotype-based method in order to simplify the experimental load of pooling single cells from multiple samples. One key rationale for doing so is that the Demuxlet requires genotyping for every input sample, in the form of a reference SNP list (ranging from 4 to 50 SNPs) for demultiplexing samples. The major innovation of this manuscript is the development of an algorithm that allows a genotyping-free experimental protocol for pooling multiple samples in the scRNA-seq results. By utilizing variational Bayesian inference, the authors directly genotyped the single cells with comparable results, though not as good in specific conditions. Despite this advantage and improvement over Demuxlet, there are some other important concerns related to the method and its application/contribution for future research.

Major concerns

1. The authors did not consider the disadvantages of NOT having a pre-defined sample information. The authors claim that they do not need to use the genotyping results, as required by the Demuxlet. However, as far as I could tell, the authors failed to address how to connect the Vireo-defined sample groups to their specific sample-of-origin. Pre-defined sample information is usually an important reference for many downstream analysis and experimental designs. Failing to connect the cell groups to specific sample donor will be of limited usage. In this scenario, Vireo is more of a ‘sample-clustering’ method instead of a ‘sample-info-recoding’ method unless the authors specifically address this point. In this aspect, the barcoding methods are much more informative as every sequence barcode corresponds to a pre-defined sample. Demuxlet does not have this problem neither, since the genotyping step generates sample-specific SNPs, which acts as a barcoding function for the defined sample.

2. Considering the advantages of barcoding tools, the manuscript lacks substantial amount of results/discussion to emphasize the utility for applying a genotyping-free algorithm Vireo using SNPs. Regarding experimental simplicity, the barcoding tools do not require genotyping, making them seem like much easier than the Demuclet protocol while keep the advantage of barcoding multiple samples. Then in terms of sample reference information (concern 1), the barcoding methods seem also have big advantage over the proposed Vireo. In addition, the SNP-based tools (both Vireo and demuxlet) have limitations in its utility for multiplexed scRNA-seq results, probably only applicable to patient samples. For example, the methods could not be used to detect the doublets of scRNA-seq across mouse models, which presumably have more homogeneous genotypes. The authors need to clearly state the advantages over existing tools.

Minor concerns:

Page 1 line 38 (right): the references should be updated since some of the barcoding methods are already published in peer-reviewed journals.

Figures 2B and 2C: Typos in the word Vireo as “Viroe”.

Figure 4D: the authors used a ribosomal protein as the example to discuss the sample-specific DE genes. However, the authors used log10(CPM+1) as a measurement for boxplots. However, according to our personal experience, these very essential genes are usually ubiquitously and highly expressed. The reason why they always jump out in DE gene analysis is because of normalization bias, e.g. TPM/CPM which is easily influenced by total UMI counts of a single cell. The authors need to consider this normalization bias in pushing RPL13 as a DE gene, unless further evidence supports the other way around.

**Authors Response**

**Point-by-point responses to the reviewers’ comments:**

Comments from Reviewer #1

Vireo aims to demultiplex pooled single-cell RNA-seq experiments by cell genotyping based on scRNA-seq reads which means that reference genotypes are not required for the input samples as long as sufficient sequencing depth is achieved to allow an order of 100 common genetic variants to be typed. The Bayesian model and inference scheme are sound and the authors tackle the problem in exactly the same way I would have - I can have no complaints. The authors provide numerical experiments to demonstrate the efficacy of the method. However, these simulations and analyses only serve to provide examples of usage. The generative model used in Vireo is a reasonable model of the physical reality and I did not find the results surprising. Methodologically the paper is of publication quality.

*We thank the reviewer for these supportive comments on our model.*

The main uncertainty in my assessment lies in the fact that the utility of Vireo is tied substantially to the pooled experimental design and its limitations. The authors end stating that "we recommend pooling 6 to 10 samples per experiment. For extremely large pools with more than twelve samples, genotype data is still required to enable accurate de-multiplexing." It is unclear to me therefore how widely Vireo would be of interest as a result.

*We thank the referee for highlighting this important point. Based on the exploration of parameters and the performance of Video (Fig. 2, Supp. Fig. S2-S3), we feel that 10 or a maximum of 12 samples can be pooled without substantial loss in accuracy. While this may be a limitation on the permitted designs, we feel that larger pool sizes are currently not desirable, as the doublet rate of existing assays (e.g. 10X genomics chromium platform) increases with the total cell count. Hence, Vireo can already be applied in the context of the vast majority of settings where pooled sample designs are warranted.*

*In the discussion section (page 6, paragraph 4-5, in blue) we explicit refer to this as a limitation, and we discuss refinements of Vireo for such settings as future work. We also highlight that the demultiplexing accuracy is intrinsically linked to the cell count per sample and sequencing depth per cell, which are two quantities that are continuing to improve. Thus, this effective limit on the pool size may increase by mere technological advances.*

Comments from Reviewer #2

Multiple groups have developed different tools to address this necessity, generating barcoding-based methods, for example cell-hashing and MULTI-seq, and

genotype-based method represented by Demuxlet. In this manuscript, the authors propose to further develop the genotype-based method in order to simplify the experimental load of pooling single cells from multiple samples. One key rationale for doing so is that the Demuxlet requires genotyping for every input sample, in the form of a reference SNP list (ranging from 4 to 50 SNPs) for demultiplexing samples. The major innovation of this manuscript is the development of an algorithm that allows a genotyping-free experimental protocol for pooling multiple samples in the scRNA-seq results. By utilizing variational Bayesian inference, the authors directly genotyped the single cells with comparable results, though not as good in specific conditions. Despite this advantage and improvement over Demuxlet, there are some other important concerns related to the method and its application/contribution for future research.

*We thank the reviewer agreed that demultiplexing of pooled samples is an important and timely task to address and its broad usage.*

Major concerns

1.	The authors did not consider the disadvantages of NOT having a pre-defined sample information. The authors claim that they do not need to use the genotyping results, as required by the Demuxlet. However, as far as I could tell, the authors failed to address how to connect the Vireo-defined sample groups to their specific sample-of-origin.

Pre-defined sample information is usually an important reference for many downstream analysis and experimental designs. Failing to connect the cell groups to specific sample donor will be of limited usage. In this scenario, Vireo is more of a 'sample-clustering' method instead of a 'sample-info-recoding' method unless the authors specifically address this point. In this aspect, the barcoding methods are much more informative as every sequence barcode corresponds to a pre-defined sample. Demuxlet does not have this problem neither, since the genotyping step generates sample-specific SNPs, which acts as a barcoding function for the defined sample.

*We thank the reviewer for this comment. To address this, we have substantially extended the discussion and now provide a more balanced recommendation in which settings Vireo should be considered in relationship to alternative strategies (page 7, paragraph 2, in blue). In particular, we feel that Vireo is most useful in settings where multiple samples are used to capture biological inter individual variation, for example as replicated in differential expression analyses; the use case we consider in Figures 3&4.*

*We now provide additional data to demonstrate that the genotypes Vireo infers are sufficient to link the samples to other omics data for which the sample identify is known (see new Supp. Fig. S12). This functionality is now also part of the software implementation and described in methods (page 10, paragraph 3). Additionally, we would like to highlight that a small number of informativ variants is sufficient to identify the pooled samples (see new Supp. Fig. S13), which can be obtained in cost-efficient manner using qPCR-based genotyping. The Vireo software can now be used to identify the most informative variants among those the high-confidence variants that have been reconstructed by the Vireo inference (see page 10, paragraph 5, in blue).*

2.	Considering the advantages of barcoding tools, the manuscript lacks substantial amount of results/discussion to emphasize the utility for applying a genotyping-free algorithm Vireo using SNPs. Regarding experimental simplicity, the barcoding tools do not require genotyping, making them seem like much easier than the Demuclet protocol while keep the advantage of barcoding multiple samples. Then in terms of sample reference information (concern 1), the barcoding methods seem also have big advantage over the proposed Vireo. In addition, the SNP-based tools (both Vireo and demuxlet) have limitations in its utility for multiplexed scRNA-seq results, probably only applicable to patient samples. For example, the methods could not be used to detect the doublets of scRNA-seq across mouse models, which presumably have more homogeneous genotypes. The authors need to clearly state the advantages over existing tools.

*We agree that molecular barcoding is an appealing complementary approach to SNP-based multiplexing. To address this comment, we have extended the discussion on this point and highlighted the utility of molecular barcoding in particular, while also clarifying the beneficial convenience of natural genetic barcodes, especially in human samples (page 12, paragraph 1, in blue). We agree with the referee that SNP-based multiplexing will be of primary interest for human samples and in the context of medical applications.*

Minor concerns:

Page 1 line 38 (right): the references should be updated since some of the barcoding methods are already published in peer-reviewed journals.

*Thanks, both references 11 and 12 have been updated.*

Figures 2B and 2C: Typos in the word Vireo as "Viroe".

*Apologies for the typos and thanks for pointing out. Both corrected.*

Figure 4D: the authors used a ribosomal protein as the example to discuss the sample-specific DE genes. However, the authors used log10(CPM+1) as a measurement for boxplots. However, according to our personal experience, these very essential genes are usually ubiquitously and highly expressed. The reason why they always jump out in DE gene analysis is because of normalization bias, e.g. TPM/CPM which is easily influenced by total UMI counts of a single cell. The authors need to consider this normalization bias in pushing RPL13 as a DE gene, unless further evidence supports the other way around.

*Thank you for this useful observation. We apologize for the lack of clarity, as the DE analysis was performed using edgeR based on the raw UMI counts and not the log10(CPM+1) values that are shown in Fig 4D to visualize the data. Instead of relying on the CPM/TPM normalization, edgeR (McCarthy, Chen, & Smyth, Nucleic acids research, 2012) employs a generalised regression model (negative binomial distribution here), which accounts for the total UMI counts per cell, as suggested by the referee. The robustness of edgeR for this type of analysis was also attested by a recent assessment of methods for DE analysis in single cells (Soneson & Robinson. Nat Methods, 2018).*

*Having said this, we agree that RPL13 is indeed not the best example to highlight. We now display results for the gene OAZ1, and display additional representative genes in Supp. Fig S6, which cover a wider range of expression quantiles.*

**Second round of review**

**Reviewer 2**

The authors have done a good job in addressing the concerns of the reviewers. I am happy to now support publication.
